# The effect of cumulative glycemic burden on the incidence of diabetic foot disease

**DOI:** 10.1186/s13018-016-0474-y

**Published:** 2016-11-18

**Authors:** Robert G. Dekker, Charles Qin, Bryant S. Ho, Anish R. Kadakia

**Affiliations:** 1 Department of Orthopaedic Surgery, Northwestern University, Chicago, IL USA; 2 Feinberg School of Medicine, Northwestern University, Chicago, IL USA; 3 Department of Orthopaedic Surgery, University of Rochester, Rochester, NY USA

**Keywords:** Cumulative glycemic burden, Diabetic foot ulcer, Charcot arthropathy, Diabetic foot disease

## Abstract

**Background:**

Glycemic control is a known modifiable risk factor for diabetic foot disease. Prior attempts to define its relationship with diabetic foot ulcer and Charcot arthropathy fail to account for variability in control and duration of diabetic disease. We developed a novel metric to reflect aggregate disease exposure in a diabetic, termed cumulative glycemic burden. We hypothesized that it would be positively associated with both diabetic foot ulcer and radiographically diagnosed Charcot arthropathy.

**Methods:**

Patients aged 18 to 90 years with ≥3 hemoglobin A1c (HbA1c) values were identified retrospectively at a single institution over a 15-year period. Primary outcomes were ICD-9 diagnosis of foot ulcer and radiographically diagnosed Charcot arthropathy. Cumulative glycemic burden was calculated by trapezoidal integration of the area under a curve defined by HbA1c values above 7 over time. Patients were stratified into quartiles based on cumulative glycemic burden (excellent, good, fair, and poor control). *χ* ^2^ tests compared the proportion of foot ulcer and Charcot across quartiles. Regression analysis identified associated demographic and comorbidity factors with diabetic foot disease. Statistical significance was set at *P* < .05.

**Results:**

Out of 22,913 diabetics, 1643 (7.2%) had a foot ulcer; 54 out of 771 diabetics (7.0%) had radiographic Charcot arthropathy. There was a statistically significant stepwise increase in the incidence of foot ulcer with increasing cumulative glycemic burden by patient quartile (5.2 vs. 6.4 vs. 7.9 vs. 13.9%; *P* < .001). No significant trend was seen between incidence of Charcot arthropathy and greater cumulative glycemic burden (7.8 vs. 5.6 vs. 4.4 vs. 10.0%; *P* = .469). Peripheral vascular disease was most strongly associated with diabetic foot ulcer. Hypertension and diabetic neuropathy were independently associated with Charcot arthropathy.

**Conclusions:**

Increasing cumulative glycemic burden is positively associated with diabetic foot ulcer. Greater attention should be paid towards the most poorly controlled diabetics with the longest duration of disease to reduce their risk. Cumulative glycemic burden is not associated with Charcot arthropathy.

## Background

It is projected that the prevalence of diabetes in the USA will nearly double in the next two decades [1]. Based on recent estimates, 15% of the growing diabetic population will develop a foot wound in their lifetime, representing a yearly $39 billion dollar healthcare expenditure [2]. If not prevented, lower extremity complications related to diabetes will become an even greater burden on the US healthcare system [3].

Diabetic foot disease is widely recognized as a significant health issue [4]. It is associated with more emergency department visits, hospital admissions, and longer lengths of stay, in addition to higher rates of amputation and premature death [5, 6]. Treatment is further complicated by the presence of multiple pre-existing comorbidities. Its substantial morbidity has led to a growing interest in strategies for earlier recognition and prevention. Still, detection often occurs too late and further investigation into the identification of at-risk diabetics is warranted [5, 7, 8].

Poor metabolic control has long been recognized to increase risk of diabetic complications, particularly diabetic foot disease [9–12]. In registry-based studies, Al-Rubeaan et al. reported that hemoglobin A1c (HbA1c), a long-term measure of hyperglycemia, was significantly higher in diabetics with foot ulcer and Stuck et al. similarly found that the incidence of Charcot arthropathy more than doubled with increasing HbA1c levels [13]. However, the relationship between diabetic control and the development of foot ulcer and Charcot arthropathy remains incompletely defined as existing studies use only a single baseline HbA1c or serum glucose level as a surrogate for total disease severity [13–15]. To our knowledge, no study has previously quantified long-term glycemic control and its relationship with diabetic foot disease.

The objective of our study was to develop a longitudinal measure of HbA1c values over time that better estimates the cumulative glycemic burden of a diabetic patient. We hypothesized that our measure would be positively associated with both diabetic foot ulcer and radiographically diagnosed Charcot arthropathy.

## Methods

The Institutional Review Board at Northwestern University approved this study prior to initiation.

### Patient population

Patients of 18 to 90 years of age with three or more documented HbA1c values over a 15-year period (2000–2014) were retrospectively identified through the enterprise data warehouse (EDW), a centralized infrastructure for unified electronic patient data at one institution. The cohort of interest was diabetic patients identified by International Classification of Diseases (ICD-9) code 250. For each patient, the total number of HbA1c values and the date that the values were collected were queried. Patients with diabetic foot ulcers were identified via the ICD-9 codes 707.1, 707.10, 707.14, 707.15, and 892.0.

### Radiographic analysis

A subset of patients was identified who met the above criteria and had at least one three-view (AP, lateral, and oblique) weight-bearing radiographic series of the foot (right, left, or bilateral) and were seen as an outpatient by either the senior author or one of two other orthopedic foot and ankle faculty. The most recent radiographic exam was accessed through the Picture Archiving and Communicating System (PACS) and examined for evidence of Charcot arthropathy. Radiographic findings of Charcot arthropathy were graded using the Brodsky classification system with the modification by Trepman et al. [16, 17]. Radiographic exams that included only one or two views of the foot, intraoperative images, or fluoroscopic images were excluded. If the patient had evidence of surgery (i.e., fusion), then the most recent pre-operative radiographic series was evaluated.

### Variables

Demographic parameters collected included age, race, gender, and body mass index (BMI). Current tobacco use or history of smoking (V15.82), hypertension (401), peripheral vascular disease (440 with all modifiers), coronary artery disease (414), chronic kidney disease (585.1–585.6), and diabetes (250) with renal (250.4), ophthalmic (250.5), and neurological (250.6) manifestations were among the comorbidities identified via their respective ICD-9 codes.

### Statistical analysis

An initial comparison between patients with and without diabetes was drawn to better understand the demographics and comorbidity profile of our cohort of interest. The two primary outcomes were ICD-9 diagnosis of diabetic foot ulcer (250.x; 707.1, 707.10, 707.14, 707.15, and 892.0) and radiographic diagnosis of Charcot arthropathy. Patients were analyzed by the presence of either primary outcome. *χ* ^2^ tests, for categorical variables, and Student’s *t* test, for continuous variables, were used to compare demographic and comorbidity profiles between cohorts.

**Fig. 1:**
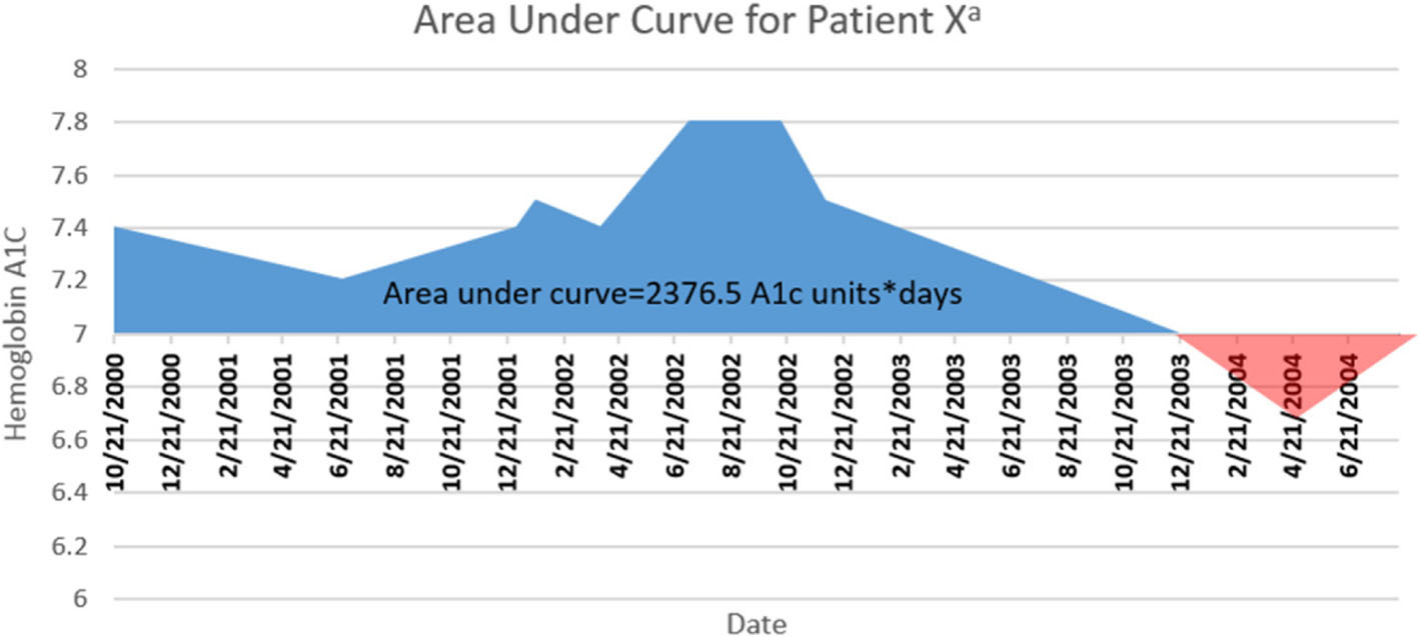
Graphical representation of trapezoidal integration for calculation of glycemic burden for a single patient

The primary study objective was the creation of a surrogate metric to quantify glycemic burden over time for diabetic patients, termed “cumulative glycemic burden”. For each patient, a curve was derived from their HbA1c levels greater than the upper limit of adequate glycemic control (HbA1c = 7) over time [18]. Each patient’s cumulative glycemic burden was calculated by trapezoidal integration of the area under the curve (AUC) above the *x*-axis as demonstrated in Fig. 1. The diabetic cohort was then stratified, via pre-determined cutoffs, into evenly distributed quartiles that represented excellent control, good control, fair control, and poor control (Table 1.) *χ* ^2^ tests compared the proportion of diabetic foot ulcer and Charcot arthropathy diagnoses among patient quartiles.

**Table 1:** Range of cumulative glycemic burden per patient quartile

Glycemic control	Cumulative glycemic burden
Excellent	<233.05
Good	233.06–781.22
Fair	781.23–2465.96
Poor	>2465.96

Secondary objectives included identifying comorbidities and patient characteristics independently associated with diabetic foot ulcer and Charcot arthropathy via regression analysis, which included risk adjustment for variables identified through univariate screening. *P* < .05 was considered statistically significant in all studies performed. All analyses were performed using SPSS version 22 (IBM Corp Armonk, NY).

## Results

### Patient population

Based on our study inclusion criteria, 33,274 patients were identified, of which 22,913 patients were diabetic as determined by ICD-9 diagnosis codes. As demonstrated in Table 2, patients with diabetes had higher rates of vascular and kidney disease, were more likely have a higher mean HbA1c, and more likely to smoke. Patients with diabetes were further stratified by the presence of diabetic foot ulcer or radiographic Charcot arthropathy. Out of 22,913 patients, 1643 had diabetic foot ulcer (7.2%). A total of 771 diabetics had foot radiographs accessible for analysis, of which 517 had X-rays of their left foot, 531 of their right foot, and 277 of their bilateral feet. A total of 54 out of 771 patients (7.0%) were found to have radiographic evidence of Charcot arthropathy. Two patients had bilateral disease. The stratification of disease by the Brodsky classification with modification by Trepman et al. can be found in Table 3.

**Table 2:** Demographics and comorbidities

Diabetic vs. non-diabetic cohort					
	No diabetes (*N* = 10,361)		Diabetes (*N* = 22,913)		*P* value
	*N*	%	*N*	%	
Gender					
Female	5442	52.6	11,901	51.9	0.306
Male	4913	47.4	11,008	48.1	
Race					<.001
American Indian or Alaskan Native	14	0.1	61	0.3	
Asian	301	2.9	863	3.8	
Black or African American	1869	18	5920	25.8	
Declined	705	6.8	1625	7.1	
Hispanic	62	0.6	152	0.7	
Native Hawaiian or other Pacific Islander	10	0.1	17	0.1	
Other	1116	10.8	3480	15.2	
Unable to answer	8	0.1	5	0	
Unknown	634	6.1	693	3	
White	5596	54	10,041	43.8	
Smoking	3769	38.2	9927	45.5	<.001
Hypertension	5774	55.7	18,569	81	<.001
Peripheral vascular disease	646	6.2	3094	13.5	<.001
Coronary artery disease	1498	14.5	7151	31.2	<.001
Chronic kidney disease	725	7	5216	22.8	<.001
Foot ulcer	133	1.3	1643	7.2	<.001
Age, year	58 (14)		62 (14)		<.001
Body mass index	31.5 (26.9)		37.2 (27.4)		0.005
HgA1c, mean	5.5 (.6)		7.4 (2)		<.001

Significance defined as *P* < .05. Continuous variables expressed as mean (standard deviation)

**Table 3:** Prevalence of Charcot arthropathy

	Type 1	Type 2	Type 3A	Type 3B	Type 4	Type 5	Total
Total	24	10	4	1	15	2	56^a^

^a^54 patients with evidence of Charcot arthropathy, 2 patients had bilateral disease

Comparisons of baseline characteristics between cohorts are documented in Tables 4 and 5. In addition to being more comorbid, the group with foot ulcers were more likely to be older (62 vs. 66; *P* < .001) and have a higher average HbA1c value (7.2 vs. 7.7; *P* < .001). No significant differences were detected between the group with and without Charcot arthropathy. The mean HbA1c values for patients with and without Charcot arthropathy were not significantly different (6.5 vs. 6.7; *P* = .401).

**Table 4:** Demographics and comorbidities

Cohort with foot ulcers vs. cohort without, diabetics only					
	No foot ulcer (*N* = 21,270)		Foot ulcer (*N* = 1643)		*P* value
	*N*	%	*N*	%	
Gender					
Female	11,204	52.7	697	42.4	<.001
Male	10,063	47.3	945	57.5	
Race					<.001
American Indian or Alaskan Native	60	0.3	1	0.1	
Asian	851	4	12	0.7	
Black or African American	5390	25.3	530	32.3	
Declined	1562	7.3	63	3.8	
Hispanic	143	0.7	9	0.5	
Native Hawaiian or other Pacific Islander	17	0.1	0	0	
Other	3204	15.1	276	16.8	
Unable to answer	4	0	1	0.1	
Unknown	678	3.2	15	0.9	
White	9307	43.8	734	44.7	
Smoking	9098	45	829	51.5	<.001
Diabetic nephropathy	2176	6.9	635	35.8	<.001
Diabetic retinopathy	3461	11	735	41.4	<.001
Diabetic neuropathy	2735	8.7	897	50.5	<.001
Hypertension	16,992	79.9	1577	96	<.001
Peripheral vascular disease	2281	10.7	813	49.5	<.001
Coronary artery disease	6149	28.9	1002	61	<.001
Chronic kidney disease	4300	20.2	916	55.8	<.001
Age, year	62 (15)		66 (13)		<.001
BMI	31.8 (7.9)		31.3 (8.9)		0.002
Mean number of HgA1c values drawn	10 (8)		11 (9)		<.001
HgA1c, mean	7.2 (1.4)		7.7 (1.6)		<.001

Significance defined as *P* < .05. Continuous variables expressed as mean (standard deviation)

**Table 5:** Demographics and comorbidities

Cohort with Charcot arthropathy vs. cohort without, diabetics only					
	No Charcot (*N* = 717)		Charcot (*N* = 54)		*P* value
	*N*	%Total	*N*	%Total	
Gender					
Female	370	51.6	26	48.1	0.624
Male	347	48.4	28	51.9	
Race					0.331
American Indian or Alaskan Native	1	0.1	0	0	
Asian	23	3.2	3	5.6	
Black or African American	179	25	14	25.9	
Declined	75	10.5	8	14.8	
Hispanic	7	1	0	0	
Native Hawaiian or other Pacific Islander	1	0.1	1	1.9	
Other	96	13.4	8	14.8	
Unknown	29	4	2	3.7	
White	306	42.7	18	33.3	
Smoking	255	38.1	21	39.6	0.828
Diabetic nephropathy	62	8.6	2	3.7	0.204
Diabetic retinopathy	96	13.4	5	9.3	0.386
Diabetic neuropathy	76	15.7	7	20	0.103
Hypertension	505	70.4	43	79.6	0.151
Peripheral vascular disease	62	8.6	4	7.4	0.754
Coronary artery disease	180	25.1	16	29.6	0.461
Chronic kidney disease	128	17.9	9	16.7	0.826
Age, year	61 (14)		57 (14)		0.087
BMI	31.7 (7.9)		31.8 (8.7)		0.301
Mean number of HgA1c values drawn	7 (6)		8 (6)		0.512
HgA1c, mean	6.7 (1.5)		6.5 (1.4)		0.401

Significance defined as *P* < .05. Continuous variables expressed as mean (standard deviation)

### Cumulative glycemic burden

**Fig. 2:**
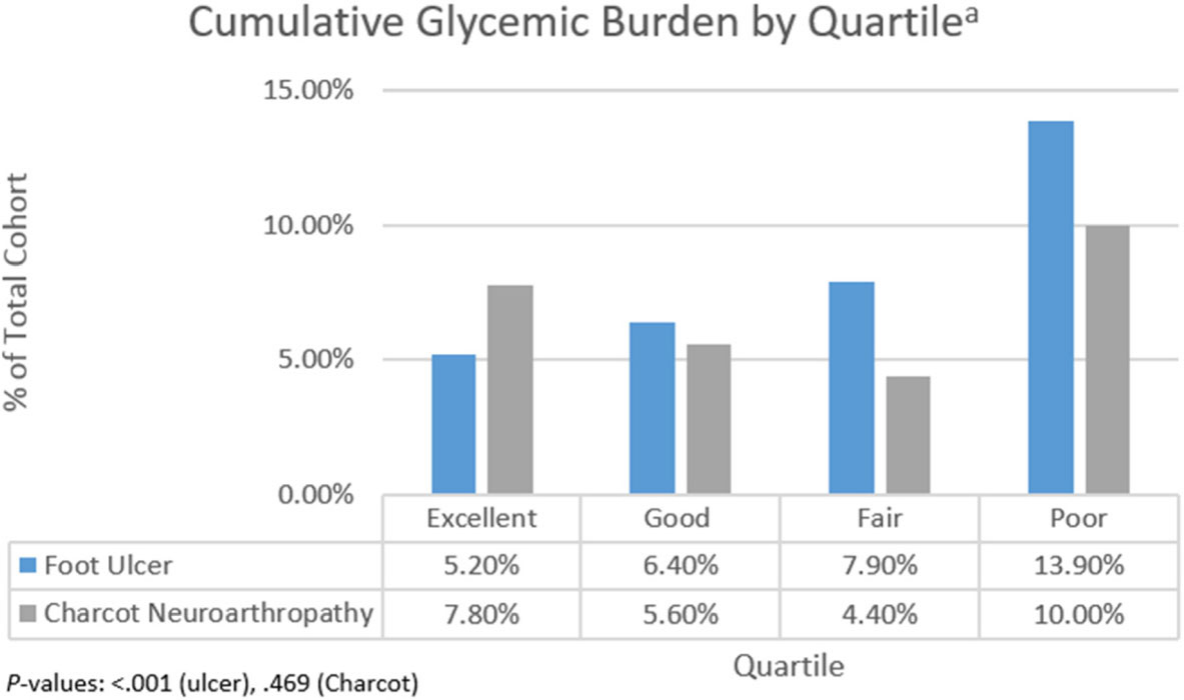
Incidence of foot ulcer and Charcot arthropathy by patient quartile

Mean cumulative glycemic burden for patients with foot ulcer was significantly greater versus patients without ulcer (2781.5 vs. 1302.8; *P* < .001). Figure 2 shows a stepwise increase in the proportion of foot ulcer diagnoses across each quartile with increasing cumulative glycemic burden in our diabetic population. The trend was statistically significant (excellent control 5.2% vs. good control 6.4% vs. fair control 7.9% vs. poor control 13.9%; *P* < .001).

Cumulative glycemic burden for patients with Charcot arthropathy was not significantly different versus patients without (1654.66 vs. 1639.83; *P* = 0.974). Similarly, no significant trend was observed across patient quartiles in the analysis of Charcot arthropathy (excellent control 7.8% vs. good control 5.6% vs. fair control 4.4% vs. poor control 10.0%; *P* = .469).

### Risk factors for diabetic foot ulcer

The strongest associated factor with diabetic foot ulcer was peripheral vascular disease (odds ratio (OR) 4.31; 3.67–5.06). The variable with the second highest odds ratio was diabetic neuropathy (OR 3.44; 2.94–4.03). Other risk factors identified in our regression model included diabetic retinopathy, hypertension, coronary artery disease, and chronic kidney disease. Increasing age was associated with lower likelihood of ulcer (OR .991; .985–.997) (Table 6).

**Table 6:** Associated risk factors of diabetic foot ulcer

	Odds ratio	95% confidence interval		*P* value
		Lower	Upper	
Age^a^	0.991	0.985	0.997	0.003
Number of A1C’s drawn	0.996	0.989	1.003	0.263
Most recent BMI	1	0.999	1.001	0.556
Diabetic retinopathy	1.357	1.154	1.595	<.001
Diabetic neuropathy	3.441	2.94	4.027	<.001
Hypertension	2.265	1.586	3.237	<.001
Peripheral vascular disease	4.309	3.668	5.062	<.001
Coronary artery disease	1.388	1.178	1.635	<.001
Chronic kidney disease	1.824	1.541	2.158	<.001

^a^Represents odds ratio for every year increase in age

### Risk factors for Charcot arthropathy

Hypertension and diabetic neuropathy were independently associated with Charcot arthropathy (OR 2.57; 1.21–4.13) and (OR 1.23; 1.04–3.04). Increasing age was associated with slightly lower risk of Charcot arthropathy (OR .964; .938–.99) (Table 7).

**Table 7:** Associated risk factors of Charcot arthropathy

	Odds ratio	95% confidence interval		*P* value
		Lower	Upper	
Age^a^	0.964	0.938	0.99	0.008
Number of A1c’s drawn	1.01	0.967	1.056	0.65
Most recent BMI	1.02	0.976	1.034	0.284
Hypertension	2.571	1.213	4.131	0.018
Diabetic neuropathy	1.233	1.035	3.038	0.049
Diabetic retinopathy	0.613	0.231	1.631	0.327
Peripheral vascular disease	0.751	0.245	2.296	0.615
Coronary artery disease	1.272	0.66	2.451	0.472
Chronic kidney disease	0.891	0.392	2.023	0.782

^a^Represents odds ratio for every year increase in age

## Discussion

Given the 5-year mortality rate for a diabetic with foot ulcer between 43 and 55%, an understanding of the diabetic disease profile is critical to prevent these devastating complications [19]. Arguably, one of the most modifiable risk factors is glycemic control, and prior attempts to define its relationship with diabetic foot disease fail to account for variability in glycemic control and disease duration. In this study, we queried the electronic medical records at a single institution over a period of 15 years for all available HbA1c values for a large diabetic cohort. Employing an area under the curve method, we developed a novel metric that may serve as a more accurate measure of lifetime glycemic exposure.

We found that 7.2% of diabetics in our cohort had foot ulcer, which is similar to previously reported rates [11, 20]. One of the most important findings of our study was that diabetics with foot ulcer not only had a significantly higher mean HbA1c but also a greater cumulative glycemic burden than those without ulcer. Previous studies have shown that hyperglycemia at one or multiple time points and duration of diabetic disease are positively associated with diabetic foot ulcer, but no study to date has accounted for varying glycemic control during the time of disease [11, 14, 15, 21, 22]. We found a statistically significant step-wise increase in the incidence of foot ulcer with worsening cumulative glycemic burden. Incidence significantly increased from excellent (5.2%), to good (6.4%), to fair (7.9%), and to poor control (13.9%) diabetics. Interestingly, the most dramatic rise occurred from the fair to poor control quartiles. This would suggest that the most poorly controlled diabetic population with longest duration of disease may warrant heightened surveillance for ulcers. To our knowledge, this is the first study to examine the relationship between lifetime diabetic disease burden and foot disease using a longitudinal measure.

A secondary objective of our study was to identify factors associated with the diagnosis of foot ulcer or Charcot arthropathy. The greatest associated factors with diabetic foot ulcers were peripheral vascular disease and peripheral neuropathy [11]. It is known that ischemia predisposes to poor wound healing, worse lower extremity function, and more frequent non-traumatic amputation in diabetics [11, 23, 24]. In agreement with a previous registry-based study, hypertension was also found to be associated with diabetic foot ulcer [11]. Age, however, was associated with fewer diabetic foot ulcers in our study [11, 15, 21, 25, 26]. Lastly, we found no significant association between diabetic foot ulcer and BMI, which is consistent with the majority of available literature [4, 22, 25, 27, 28]. Still, there is a lack of consensus in the literature of the relationship between age, BMI, and diabetic foot ulcer [29]. Additional studies are required to more clearly define this relationship.

Of the patients who met our inclusion criteria for radiographic analysis, 7% had Charcot arthropathy. Previous studies report similar rates in diabetic populations, ranging from 0.8 to 7.5% [21]. Cumulative glycemic burden was not statistically greater in patients with Charcot arthropathy, a finding likely reflective of the observation that both the group with and without Charcot arthropathy diagnoses had rather adequate glycemic control with average HbA1c values of 6.7 and 6.5, respectively.

A unique finding in our study was the positive independent association between hypertension and Charcot arthropathy. While this finding may be purely incidental as diabetics often suffer from multiple comorbidities, hypertension may be a surrogate for a pro-inflammatory state, which has been shown to contribute to the pathogenesis of Charcot arthropathy [30]. Lastly, increasing age was associated with lower incidence of Charcot arthropathy. Previous studies have found an increased incidence in diabetics over the age of 60 and an association with longer disease duration, while others show no association with age [13, 14]. Our findings with regard to age, while meeting statistical significance, are likely not clinically significant, and thus, heightened surveillance for Charcot arthropathy should be maintained for diabetics in all age groups. A higher incidence in younger patients would however support a neurotraumatic etiology for Charcot arthropathy as younger, more active individuals would be more prone to repetitive micro-trauma. However, we were unable to measure activity level in our cohort in order to support this hypothesis; thus, further studies are warranted.

Our study must be interpreted in the context of its limitations. We collected and analyzed data retrospectively for a large intra-institutional sample size of over 20,000 patients. Given the retrospective nature of our study, we cannot draw any conclusive causal relationships particularly in the absence of known timing of diagnoses and linear follow-up of subjects. Conclusions drawn through univariate analysis should be interpreted cautiously given the numerous confounding factors. As with all observational studies, patient demographic, comorbidities, and diagnoses were collected using diagnostic codes from medical records. These may not accurately represent patients’ actual clinical status; however, all associated conditions found are well recognized in the literature. While the use of ICD-10 coding may maximize usefulness to future generations, our data was selected with ICD-9 coding in order to collect a robust population of patients.

Patients with and without a diagnosis of foot ulcer or Charcot arthropathy may fall into different defined quartiles of glycemic control at different times, which may over- or underestimate the associations uncovered in our study. The fluctuating nature of glycemic control requires a fluid metric, such as cumulative glycemic burden, that accounts for both time and change in HbA1c. Moreover, it is possible that variability in the precision of the trapezoidal integration method, which was used to calculate cumulative glycemic burden, exists due to inclusion of patients with at least three recorded HbA1c values, but no maximum limit. However, the difference in mean number of HbA1c values available for patients with and without the presence of foot ulcer (11 vs. 10), as well as with and without the presence of radiographic Charcot arthropathy (8 vs. 7), was not clinically significant. It is also possible that variability in patient follow-up may have affected measured values of cumulative glycemic burden. We therefore acknowledge that our measure, as applied in this investigation, is subject to some degree of error.

For our radiographic analysis, the inclusion of diabetics who at one time had been evaluated in an outpatient orthopedic foot and ankle clinic creates selection bias, which may have led to an overestimation of the incidence of Charcot arthropathy in our diabetic population. Acute cases diagnosed solely on non-radiographic, clinical parameters (presence of swelling, erythema, warmth, etc.) may have been missed due to our methodology.

Finally, interventions upon diagnosis of the foot ulcer including off-loading and casting and the compliancy of subjects in optimizing their glycemic control are unknown. The authors emphasize that the objective of the study was to simply determine the association between cumulative glycemic burden and diabetic foot disease incidence.

## Conclusions

Increasing cumulative glycemic burden is positively associated with diabetic foot ulcer. Greater attention should be paid towards the most poorly controlled diabetics with the longest duration of disease to reduce their risk of diabetic foot ulcers. Cumulative glycemic burden is not associated with Charcot arthropathy.

## Abbreviations

AUC: Area under the curve
BMI: Body mass index
EDW: Enterprise data warehouse
HbA1c: Hemoglobin A1c
ICD: International Classification of Diseases
OR: Odds ratio
PACS: Picture Archiving and Communicating System
